# In Vitro Detection of Acaricide Resistance in Hyalomma Species Ticks with Emphasis on Farm Management Practices Associated with Acaricide Resistance in Abu Dhabi, United Arab Emirates

**DOI:** 10.3390/vetsci12080712

**Published:** 2025-07-29

**Authors:** Shameem Habeeba, Yasser Mahmmod, Hany Mohammed, Hashel Amer, Mohamed Moustafa, Assem Sobhi, Mohamed El-Sokary, Mahmoud Hussein, Ameer Tolba, Zulaikha Al Hammadi, Mohd Al Breiki, Asma Mohamed Shah

**Affiliations:** 1 Biosecurity Affairs Division, Development and Innovation Sector, Abu Dhabi Agriculture and Food Safety Authority (ADAFSA), Abu Dhabi P.O. Box 52150, United Arab Emirateszulaikha.alhammadi@adafsa.gov.ae (Z.A.H.); mohammed.albreiki@adafsa.gov.ae (M.A.B.); asma.mohammed@adafsa.gov.ae (A.M.S.); 2 Department of Veterinary Clinical Sciences, College of Veterinary Medicine, Long Island University, 720 Northern Boulevard, Brookville, NY 11548, USA; 3 Applied Research & Capability Building Division, Abu Dhabi Agriculture and Food Safety Authority, Abu Dhabi P.O. Box 52150, United Arab Emiratesameer.tolba@adafsa.gov.ae (A.T.); 4 Extension Services & Animal Health Division, Animal Wealth Sector, Abu Dhabi Agriculture and Food Safety Authority (ADAFSA), Abu Dhabi P.O. Box 52150, United Arab Emirates; mohamed.abdulhalim@adafsa.gov.ae (M.M.); assem.abdelazeem@adafsa.gov.ae (A.S.); 5 Section of Veterinary Sciences, Health Sciences Division, Al Ain Men’s College, Higher Colleges of Technology, Al Ain 17155, United Arab Emirates; melsokary@hct.ac.ae; 6 College of Veterinary Medicine, University of Al Dhaid, Sharjah P.O. Box 27272, United Arab Emirates

**Keywords:** acaricide resistance, Abu Dhabi, farm management practices, in vitro bioassay, ticks, control measures

## Abstract

Acaricide usage has led to the growing problem of resistance in ticks. A heavy tick burden and the presence of ticks on animals throughout the year, despite the monthly application of acaricides, in farms in the United Arab Emirates formed the motivation for this study. The objectives of this research were as follows: (a) to assess the acaricide resistance status of the most prevalent tick *Hyalomma* spp. to widely used acaricides Cypermethrin and Deltamethrin; (b) to identify the association of farm management practices and farm-level risk factors with the failure of tick treatment (acaracide resistance). A total of 1600 ticks were collected from 20 farms located in three different regions of Abu Dhabi Emirate including Al Ain (*n* = 10), Al Dhafra (*n* = 5), and Abu Dhabi (*n* = 5). The ticks were subjected to an in vitro bioassay adult immersion test (AIT) modified with a discriminating dose (AIT-DD) against commercial preparations of Cypermethrin and Deltamethrin. A questionnaire was designed to collect metadata and information on farm management and the farm-level risk factors associated with routine farm practices relating to the treatment and control of tick and blood parasite infections in camels and small ruminant populations. *Hyalomma anatolicum* and *Hyalomma dromedarii* were identified among the collected ticks, with *H. anatolicum* being the most prevalent tick species (70%) in the present study. The test results of the in vitro bioassay revealed varied emerging resistance to both of the acaricides in the majority of the three regions; fully susceptible tick isolates with zero resistance to Deltamethrin were recorded in one farm at Al Ain and two farms in the Abu Dhabi region. A questionnaire analysis showed that the failure of tick treatment in farms varied with the presence or absence of vegetation areas, types of animal breeds, and management practices. This study reports the emergence of resistance in ticks to Cypermethrin and Deltamethrin across the Abu Dhabi Emirate, indicating a strict warning for the cautious use of acaricides. There is also a need to improve awareness about sound tick management and control practices among farm owners through a multidisciplinary approach adopting integrated pest management strategies that engage farmers, veterinarians, and policy makers.

## Simple Summary

Ticks are blood-feeding ectoparasites with a global distribution; they serve as vector reservoirs for the spread of several pathogens among their vertebrate hosts. Even though tick control is advocated through integrated tick management programs, the use of chemical acaricides remains the mainstay against ticks. Acaricide treatments are now facing increasing failures due to the development of resistance against commonly used drugs like synthetic pyrethroids. This study reported the resistance of *Hyalomma* spp. ticks to commercial preparations of Deltamethrin and Cypermethrin in three different regions of the Emirate of Abu Dhabi, United Arab Emirates. A questionnaire survey was conducted with the farm owners in the region to generate baseline data about the knowledge, attitude, and practices of farmers regarding ticks and tick-borne diseases. The findings from this study will help to design awareness programs about ticks and tick-borne diseases, as well as improving the adoption of effective tick prevention and control measures in the region.

## 1. Introduction

Ticks are voracious blood-feeding ectoparasites that transmit pathogens; they have a negative impact on the livestock industry, resulting in severe economic losses, estimated to be USD 18.7 billion annually [1]. Considerable efforts are invested in the chemical control of these ectoparasites using highly toxic acaricides, which has been the main strategy for tick control for decades, resulting in acaricide-resistant strains. The climate and vegetation of Middle Eastern and MENA (Middle East and North Africa) countries provide a suitable habitat for tick survival [2]. The United Arab Emirates (UAE) has a significant wealth of livestock, with the total number of camels being estimated at 450,000, while the total number of sheep and goat herds is estimated at about 5 million, thus constituting an important component of both food security and the national economy. These animals produce considerable amounts of milk, meat, wool, and hides [3], while ticks constitute a major threat to the livestock industries in the MENA region. Thousands of livestock are imported annually from Sudan, Somalia, Turkey, Iran, Uruguay, Pakistan, Australia, and Argentina to Saudi Arabia and the UAE [4]. Thus, local farms have a frequent input of imported livestock including cattle, sheep, and goats, allowing the local population of ticks to feed on naïve hosts from outside the region, enhancing the tick population density and evolution. About 55 species of ticks belonging to eight genera have been documented in the MENA region, including two genera of soft ticks and six genera of hard ticks [2]. Among these, *Hyalomma* and *Rhipicephalus* are the most common genera reported from livestock in almost all Arab countries. Even though documented records on tick-borne diseases are less in the UAE, the presence of *Theileria annulata* and spotted fever group Rickettsia sp. was recorded in camel ticks in the UAE [5]. Tick control using chemical acaricides is now facing challenges due to the presence of populations that are resistant to major classes of acaricides. The indiscriminate use of acaricides for the control of ticks by livestock owners, as well as the presence of high tick loads on camels over the entire year, in spite of the monthly application of acaricides, has been reported in the Al Ain region of the United Arab Emirates [6]. Limited studies have been conducted on acaricide resistance in the UAE, with a single previous report of Cypermethrin resistance being recorded in *Hyalomma anatolicum* Koch 1844 ticks in Al Ain, UAE [7], using an adult immersion test. A diagnosis of resistance against different acaricides is essential and urgently required through efficient and inexpensive tests that can be easily followed [8]. Amongst the various bioassays proposed for the determination of acaricide resistance in ticks, the adult immersion test with a discriminating dose (AIT-DD) is the most rapid and simple method for the large-scale screening of field ticks [9]. The tick infestation and treatment failures reported in the Emirate warrants resistance studies to be conducted in a periodic manner in order to monitor the status of acaricide resistance in the tick population.

Even though climate change impacts, including higher mean temperatures, increased humidity, the frequent influx of imported livestock, demographic modifications, and many other factors, facilitate the survival and establishment of tick colonies in the region, the fundamental solution to the problem lies in the scientific management of farm activities including tick control practices. The periodic monitoring of the resistance status of the tick population to available acaricides, along with an understanding of the tick control practices followed by the farm workers, is pivotal in identifying the critical gaps in tick control. Systematic studies on acaricide resistance development in tick populations are still in their infancy in the region, highlighted by a lack of publications. Thus, the present study aimed to (a) assess the acaricide resistance status of the most prevalent tick *Hyalomma* spp. to widely used acaricides Cypermethrin and Deltamethrin, and (b) identify the association of farm management practices and farm-level risk factors with the failure of tick treatments (acaracide resistance).

## 2. Materials and Methods

### 2.1. Study Area

The United Arab Emirates (UAE) is located in the oil-rich and strategic Arabian or Persian Gulf region. It adjoins the Kingdom of Saudi Arabia and the Sultanate of Oman. The Emirate of Abu Dhabi is one of the seven Emirates that constitute the United Arab Emirates and is the largest Emirate, accounting for 87% of the nation’s total land area (67,340 km^2^). Abu Dhabi is located in the far western and southwestern part of the United Arab Emirates along the southern coast of the Persian Gulf between latitudes 22°40′ and around 25° north, as well as longitudes 51° and around 56° east. The topography of the Emirate is dominated by a low-lying sandy terrain dotted with sand dunes exceeding 300 m (980 ft) in height in some southerly areas. Land cultivation and irrigation for agriculture and forestation over the past decade has increased the size of the green areas in the Emirate to about 5% of the total land area, including parks and roadside plantations. The Emirate’s high summer (June to August) temperatures are associated with a high relative humidity. The Emirate is divided into three municipal regions—the Abu Dhabi Region, the Al Dhafra Region (Western Region), and the Al-Ain Region (Eastern Region). Fully engorged adult female ticks were collected from naturally infested livestock (cattle/camel/sheep/goat) from the selected farms that had tick infestation problems with observed tick treatment failures from the three regions of Abu Dhabi. Ticks were removed from the body of each examined animal manually using forceps and were placed in 50 mL plastic vials. A total of 1600 ticks were collected in ventilated vials from different selected farms; these were closed with a muslin cloth to allow air and moisture exchange and were brought to the laboratory in cardboard containers that were kept in a cool box, which was transported to the laboratory on the same day as collection. Only engorged female ticks weighing an average of 250 mg were selected, whereas damaged and discolored ticks were rejected. A bioassay was conducted within 24 h of collection from the animals. Ticks were identified to the species level via microscopy, using taxonomic keys as per standard references [10].

### 2.2. Acaricide Resistance Studies

An in vitro bioassay—the adult immersion test (AIT)—modified with a discriminating dose (AIT-DD) was conducted.

A bioassay for assessing the resistance against synthetic pyrethroids (Cypermethrin and Deltamethrin) was performed in *Hyalomma anatolicum*. A commercially available preparation of Cypermethrin (10.25% EC) and Deltamethrin (10 mg/mL Pour On,) was diluted in water to prepare the respective discriminating concentrations, as per standard references [9].

For the experimental bioassay, AIT-DD, discriminating doses of the acaricides were prepared in distilled water from the stock solutions and were tested against adult engorged female *Hyalomma* spp. The adult immersion test with discriminating doses (AIT-DD) was conducted in triplicate, along with control ticks, and the results were recorded as an average of the three replicates against the tick species *Hyalomma anatolicum*.

The bioassay was conducted according to the standard methods with slight modifications [9]. The following procedure was adopted for AIT-DD: The discriminating dose for Cypermethrin was 489.74 ppm [7] and for Deltamethrin was 75 ppm [11]. Ten healthy, engorged female ticks were immersed in 20 mL of the freshly prepared acaricide dilution for 30 min at room temperature, along with the control group, which was immersed in water, with gentle and intermittent shaking. After 30 min, the acaricide solution was poured off and the ticks were dried gently on filter paper. Three replicates of ten ticks were used for the testing of each acaricide, along with the controls. After that, the ticks were stacked, ventral side up, onto double-sided sticky tape in a Petri dish, before being placed in a constant climate chamber (Memmert HPP) (85 to 95% RH, 28 ± 2 °C) for 7 days. After 7 days, the ticks from the treatment and control groups were observed, and the number of ticks that laid eggs were counted and recorded.

Ticks that were treated with acaricide but still laid eggs were considered as being resistant, while ticks that were treated with acaricide and did not lay eggs were considered as being susceptible.

The percentage resistance was calculated as per the following formula: R (%) = (Nt/Nw) × 100, where R (%) refers to percent resistance, Nt refers to the number of treated ticks laying eggs, and Nw refers to the number of untreated ticks laying eggs [9,11,12,13]. In this study, the ticks were considered to be resistant if the percentage resistance (%R) was greater than 80% on the seventh day after incubation. They were considered to be susceptible if they laid less than 80% of the eggs laid by the control [12].

### 2.3. Questionnaire Survey

A questionnaire was designed to collect information about farm management and the farm-level risk factors associated with tick infestation; tick-borne diseases, the frequency, type, and mode of acaricide treatment adopted by the owners; and owners’ experiences relating to the efficacy of commonly used acaricides. To cover all farms in Abu Dhabi, fifteen farms were selected in Abu Dhabi (6), Al Ain (4), and Al Dhafra (5) in order to ensure a good representation of the study population. Ticks were collected from the same farm animals and surrounding environments to investigate the development of tick resistance to acaricides.

### 2.4. Statistical Analyses

A binary outcome variable was created to represent whether acaricide treatment for tick control had failed (Yes or No). The model included several explanatory variables (potential risk factors), such as geographic region, the co-housing of animals, vegetation surrounding the farm, the method of acaricide application, feeding practices, the presence of tick infestation, animal breed and species, season, treatment approach for tick infestation, acaricide use strategy, application technique, tick prevention measures implemented on the farm, and the specific drug used for treatment.

Initially, descriptive statistics were used to summarize and explore the distribution of the potential risk factors. A logistic regression analysis was then employed to examine the relationship between acaricide treatment failure and the identified risk factors. The regression model was developed using the “glm” function with a binomial distribution in R (version 3.3.3) [14]. A univariable logistic regression was performed first to assess the unadjusted associations between the outcome and each explanatory variable, as well as to identify candidates for inclusion in the multivariable analysis. Variables with a *p*-value ≤ 0.20 were considered for the multivariable model [15]. The final model was obtained through backward stepwise elimination, sequentially removing the least-significant variables until only those with a *p*-value ≤ 0.05 remained. For each variable retained in the final model, the *p*-value, odds ratio (OR), and 95% confidence interval (CI) were reported. Statistical significance was determined at a threshold of *p* ≤ 0.05 across all analyses.

## 3. Results

### 3.1. Tick Identification Studies

The present study identified only a single genera of ticks, i.e., Hyalomma spp. Among the *Hyalomma* ticks that were dominant in the investigated area for the present study, *Hyalomma anatolicum* Koch 1844 and *Hyalomma dromedarii* Koch 1844 were speciated based on various morphological characteristics, including the length of mouth parts, the number of festoons, punctuations on the scutum on the dorsal side, spurs on the pedipalp segments, and other specific features that were revealed using light microscopy [10]. *Hyalomma anatolicum* was the most prevalent tick species (70%) in the present study and was used for the bioassay.

### 3.2. Acaricide Resistance Studies

The results of the adult immersion test with discriminating doses for Cypermethrin and Deltamethrin of 489.74 ppm and 75 ppm, respectively, are recorded in Table 1. The AIT-DD revealed a resistance of more than 80% to Cypermethrin and Deltamethrin in Malaqet, Al Ain region (Table 1), where the ticks can be highly resistant.

The percentage of resistance against Cypermethrin ranged from 20 to 100% in *Hyalomma* spp. ticks, with highest resistance observed in the camel farms of the Malaqet area of the Al Ain region. The percent resistance of tick isolates against Deltamethrin ranged from being fully susceptible to its highest at 90% in *Hyalomma* spp. collected from camels from the Malaqet area in the Al Ain region. Even though the AIT-DD revealed varied emerging resistance to both the acaricides (Cypermethrin and Deltamethrin) in the majority of the three regions (Table 1b,c), fully susceptible tick isolates with zero resistance to Deltamethrin were recorded in Sia Sabra (Al Ain), as well as in two farms in the Abu Dhabi region. In the present study, the ticks collected from camel and sheep farms located in the Malaqet area (Al Ain) recorded 100% and 90% resistance against Cypermethrin. The results revealed varying levels of emerging resistance in *Hyalomma* spp. to Cypermethrin and Deltamethrin in tick populations from all three regions in the Emirate of Abu Dhabi.

### 3.3. Questionnaire Survey Analysis

A description of the study population, farm characteristics, and the management practices, including their level and frequency, is presented in the Supplementary Material. The survey included a total of 15 farms in Abu Dhabi (*n* = 6), Al Ain (*n* = 4), and Al Dhafra (*n* = 5). The animal species and production types in this study were small ruminants (*n* = 5), mixed production (*n* = 3), and camels (*n* = 7). The majority of the farm owners kept their animals indoors and together (*n* = 13), while the farm laborers work and reside on the farm premises itself (*n* = 14). The majority follow a stall-feeding system for feeding management. All the farms reported 100% tick infestation, with 13 farms reporting a chronic infection of ticks. Most farms applied acaricide on an irregular basis (*n* = 11). Unfortunately, the majority of the farm owners and workers (*n* = 12; 80%) were unaware of tick resistance as an emerging animal health problem. Most of the farm owners and workers (*n* = 9) did not know that a sandy floor is a risk factor for tick presence.

The majority of the farm owners and workers (*n* = 14) know that the hot season has the highest risk for tick infestation. When tick infestation is observed, some farmers call the veterinarian (*n* = 5) as their first choice, while others use herbal/traditional treatments (*n* = 8). It was also noted that all the farms in the study had neither a proper plan for acaricide use nor a proper disposal procedure for used acaricide bottles. Most of the farmers (*n* = 12) used spraying/dipping as a preferable method of using acaricides. Additionally, most farmers (*n* = 13) did not treat animal houses and premises with acaricide, which increases the risk of tick infestation and persistence on the farm. Most farmers (*n* = 10) experienced the problem of tick treatment failure. Our study revealed the failure of tick treatment was 2.23 times higher on farms with marshy/sandy areas compared to those without these areas. The failure of tick treatment in indigenous animal breeds was reduced by 3.58 compared to the crossbred animals. When tick treatment failure occurred, some of them repeated the treatment (*n* = 4), others increased the dose (*n* = 7), and the rest consulted a veterinarian (*n* = 3). Most of the farmers (*n* = 10) used an alternative combination treatment with Cypermethrin and Ivermectin. The majority (*n* = 14) of the farms reported the incidence of tick-borne diseases Theileriosis and Babesiosis (*n* = 1). The identified risk factors, as per the questionnaire survey study, that are highly associated with the failure of acaricide treatment include the following: (a) a lack of awareness from farmers relating to the application of acaricides, (b) poor farm management with the crowding of animals and a lack of ideal flooring, and (c) irregular and repeated use of acaricides, resulting in resistant tick populations.

The associations between the failure of tick treatment using acaricides and some management factors are shown in Table 2. Our findings indicate that the failure of tick treatment in farms surrounded by vegetation areas has a higher odd by 2.23 compared to farms without vegetation areas. This indicates that the vegetation areas are a good habitat for ticks and tick-borne disease hosts. Therefore, it is highly recommended to remove vegetation from the farm and clean the surrounding environment. The risk of the failure of tick treatment in indigenous animal breeds is 3.58 less compared to the crossbred animals. This is a strong indication that local breeds of camel and small ruminant are resistant to infection with ticks and the failure of tick treatment, instead responding well to the tick treatment and use of acaricides.

## 4. Discussion

The *Hyalomma* species is identified as the dominant tick species in the Middle East [16]. *Hyalomma* ticks are major parasites of domestic animals and efficient vectors of a variety of disease-causing pathogens including Crimean Congo Hemorrhagic fever virus (CCHF), as well as different species of *Theileria* viz., *T. annulata*, *T. lestoquardi*, *T. ovis*, *T. equi*., rickettsial pathogens viz., *R. aeschlimannii*, *C. burnetii*, *R. conorii*, *R. sibirica*, *R. africae*, *A. phagocytophilum*, *A. marginale*, *A. ovis*, *A. centrale*, *Coxiella burnetti*, and *Ehrlichia canis* [17]. *Hyalomma* and *Rhipicephalus* are the most common genera reported on livestock in almost all Arab countries, being particularly widely distributed in the MENA region [2]. In neighboring countries like Iran, Turkey, Iraq, Oman, Saudi Arabia, Kuwait, Yemen, Qatar, and Bahrain, the most abundant collected ticks from livestock were from the *Hyalomma* genus [16,18,19].

*H. anatolicum* is the most widespread species, parasitizing cattle, camel, sheep, and goats. It is considered as the main vector for ovine and caprine malignant Theileriosis and tropical Theileriosis, which are caused by *Theileria lestoquardi* and *Theileria annulata,* respectively. In the present study, we identified only *Hyalomma anatolicum* and *H. dromedarii*, considering the regional importance of documented cases of CCHF in humans and animals in the region [20], along with increasing cases of Theileriosis in farm animals; as such, *H. anatolicum* was used for the bioassay. Differentiating engorged females of *H. anatolicum* and *H. dromedarii* is often challenging, but morphological features like longirostrate mouth parts; less-prominent cervical grooves; and distinct leg spurs on coxae 1, 2, and 3 help identify *H. anatolicum*. When morphological features are unclear, molecular methods can be used for species confirmation; however, this was not attempted in this study. With little published data on any reference tick strains; no country-specific bioassays, or biochemical and molecular assays; and no systematic work on resistant characterization, the study utilized AIT-DD for assessing the percentage-based resistance classification. Moreover, a country-specific discriminating concentration (DC—two times the concentration that would kill 99% of ticks that are susceptible to the molecule) was available only for *H. anatolicum* [7], which is useful for monitoring changes in the resistance status of ticks in this Emirate. Continued validation studies are required to obtain discriminating doses for different species of ticks on different acaricides, as well as to establish country-specific acaricide-susceptible reference tick isolates.

In the present study, the ticks collected from camel and sheep farms located in the Malaqet area (Al Ain) recorded 100% and 90% resistance against Cypermethrin, which is in agreement with earlier findings from Habeeba et al. [7], where level II resistance was reported in the Malaqet region (Al Ain). Resistance in ticks was classified as susceptible (RF ≤ 1.4), level I resistant (RF = 1.5–5.0), level II resistant (RF = 5.1–25.0), level III resistant (RF = 25.1–40), or level IV resistant (RF > 40.1) based on resistance factors in reference to a susceptible tick reference strain [21]. The present data demonstrate comprehensive information on the level of resistance of multi-host tick *H. anatolicum* to commonly used synthetic pyrethroids using the in vitro bioassay AIT-DD [22]. The increased expression of detoxification enzymes was found to be a possible reason for resistance development in *H. anatolicum* ticks collected from different districts of Haryana, India. Further studies are warranted to explore the underlying mechanism of resistance, including metabolic detoxification, target site insensitivity, and genetic mutations in the tick populations.

The development of Cypermethrin and Deltamethrin resistance against different species of ticks has been reported worldwide, including in Africa, South America, Central America [1], and, as reported in this study against *Hyalomma* spp., Asia [12,13,23,24]. Similar results with level III Cypermethrin resistance in *Hyalomma anatolicum* were reported in the Al Hamam region (Al Ain) and level II resistance was observed in the Malaqet region (Al Ain), whereas level I resistance was observed in the Abu Dhabi and Al Dhafra regions, other than Sweihan (Al Ain), where the tick population was fully susceptible [7].

The inappropriate repeated usage of Deltamethrin and Cypermethrin in the regions of this study might be one cause relating to the development of resistance against ticks. *H. dromedarii* ticks were reported to be found on camels in a private farm in Al Ain over the entire year, despite the monthly application of an acaricide [6], suggesting either the inadequate use of the acaricide or the development of acaricide resistance. One of the most important factors that affects the efficacy of an acaricide is the use of an acaricide with an incorrect concentration; this is a major cause of tick control failure at communal dipping tanks [25,26]. The failure to control ticks despite acaricide application leads to either an increase in the concentration of the acaricide or its repeated usage at short intervals with a gap of 3 days. Increasing the acaricide concentration leads to a higher selection pressure for tick resistance, as higher acaricide concentrations effectively kill all susceptible ticks, leaving only highly resistant individuals in the population [27]. Each successive use of higher concentrations, in a selective process, would concentrate the genes responsible for the resistance, and, eventually, the majority of the ticks in the population would be resistant to the acaricide being applied against them [27,28]. The prolonged use of synthetic pyrethroids has probably contributed to the resistance of tick populations in the region. Deltamethrin and Cypermethrin were actively in use in the study areas. Stringent regulations on acaricide use can limit the availability of other effective products. Our questionnaire analysis showed the lack of knowledge of farmers in relation to the scientific management and control of ticks, including the use of chemical acaricides. This approach suffers many drawbacks including the repetition of drug application in an irregular manner, as well as the use of high doses of acaricides that will result in the emergence of resistant populations of ticks. This is a very important finding that identifies unscientific farming activities as the fundamental problem that triggers acaricide resistance and tick treatment failures. It was concluded that the use of acaricides at high frequencies and high concentrations was one of the main causes of tick resistance in the study areas. In addition, extreme heat and arid conditions in the desert can lead to the increased evaporation and degradation of acaricides, reducing their residual activity. Climate constraints and remote or isolated farming areas are challenges that disrupt the supply chain and availability of acaricides. Acaricide resistance reports in the present study, along with survey analysis reports, warrant immediate interventions such as the improved education and awareness of farm owners in relation to the strict and cautious use of acaricides, as well as the adoption of integrated tick management strategies. Our present study detects phenotype resistance in ticks by recording the percentage of survival at discriminating doses; however, monitoring the genotype/resistant alleles has to be carried out in a systematic way in order to confer the correlation between phenotypic and genotypic changes in these tick populations.

Large numbers of animals imported into the region not only serve as a source of pathogens, but also as a source of a resistant tick population. The occurrence of *H. dromedarii* ticks on camels in vicinities in the UAE that are on the border with Oman, such as Al-Wagan, Omghafa, Malaket, Mezyad, and Dawar Al-Shahenat, may result in a cross-border movement of the ticks between the two countries [29]. Our study reported a highly resistant type of tick in the Malaqet area, Al Ain, which shares a border with Oman. Cross-border animal movement should be better monitored in order to understand the introduction of acaricide-resistant alleles among tick populations.

Local farms can provide favorable conditions for ticks to breed and spread because they provide a habitat with relatively high moisture that is sheltered from the harsh desert environment [29]. *Hyalomma* ticks are two- or three-host ticks and remain attached to the host only for blood feeding, spending the majority of their lifetime outside the host. Local farms in the United Arab Emirates have suitable environments for ticks, with sand beds/sand flooring in which ticks can burrow a few centimeters below the ground to find both favorable microhabitats for egg deposition and refuge from chemical spraying. These factors play a large role in permitting these ticks to exist and even thrive in escaping the acaricides or natural predators. The failure of tick treatment in farms with sandy areas and vegetation was found to be 2.23-fold higher compared to farms without sandy areas. This indicates that the marshy/sandy areas are a good habitat for the ticks and, therefore, tick-borne diseases. The plastering of floor surfaces and walls with smooth cement also helps avoid shed infestation by removing the potential hiding places of some tick species (such as *Hyalomma*) that hide in cracks and crevices [30].

Our research indicated that the failure of tick treatment in indigenous animal breeds was found to be lower than that of crossbred animals. This highlights a significant difference in tick resistance between these groups. Indigenous breeds often exhibit a higher level of resistance to parasites like ticks due to their evolutionary adaptation to local environmental conditions, which enhances their natural immunity. This natural resistance allows them to better withstand tick infestations and respond more effectively to treatment compared to crossbred animals. Crossbred animals, while often bred for traits like increased productivity, may lack this inherent resistance, making them more susceptible to tick infestations and less responsive to treatments [26,27,31].

Previous studies have shown that indigenous cattle breeds tend to have fewer and less-severe tick infestations than their crossbred counterparts, likely due to their genetic resistance to parasites [32]. It was reported that the indigenous breeds of sheep and goats are more resilient to ticks due to traits such as thicker skin, effective grooming behaviors, and a more robust immune response [33]. These traits have evolved through natural selection, allowing indigenous breeds to survive and thrive in areas with a high tick pressure. Crossbred animals, on the other hand, often lack these adaptive traits, making them more susceptible to both tick infestations and treatment failure [34].

Indigenous camel breeds often exhibit physical and behavioral traits that enhance their ability to resist tick infestations, which help reduce the tick burden. Research on indigenous livestock has consistently shown that native breeds are more resilient to parasitic infections compared to crossbreeds, whose genetic diversity might compromise their ability to develop strong resistance mechanisms [35]. Crossbred camels, typically bred for improved productivity traits, may sacrifice some of the innate resistance to parasites that indigenous breeds possess, making them more vulnerable to tick infestations and resulting in higher rates of treatment failure [36]. This suggests that the genetic selection of camels for traits other than parasite resistance may be a factor in the reduced effectiveness of tick treatments in crossbred camels. The reduced failure rates of tick treatments in indigenous animals suggest that breeding programs focused on maintaining or enhancing these natural resistances could be a more sustainable approach to managing tick infestations in small ruminants.

Our findings showed that the failure of tick treatment in farms surrounded by vegetation areas has 2.23-fold higher odds compared to farms without vegetation areas; this can be explained by the ecological dynamics of tick populations. Vegetation-rich areas provide ideal habitats for ticks to thrive, offering moisture, shelter, and hosts (such as wildlife and rodents) that facilitate the lifecycle and spread of ticks. This increases the likelihood of re-infestation and can contribute to the reduced efficacy of tick treatments. The tick infestation rate is maximized in spring and summer when there is suitable vegetation in pastures for grazing livestock [18]. Previous studies have shown that ticks are more abundant in regions with dense vegetation areas because environments with shaded micro-climates increase local questing tick numbers and support their survival and reproduction [37,38,39]. Additionally, vegetation may harbor wildlife reservoirs that act as alternate hosts for ticks, maintaining a persistent source of infestation on farms [40]. On farms without vegetation, tick populations are often lower due to the less-hospitable conditions, making tick control measures more effective. The reported increased odds (2.23 times) of treatment failure in vegetated areas underscore the importance of integrated tick management strategies that account for environmental factors, including habitat modification, in addition to chemical treatments.

## 5. Conclusions

This study reports the emergence of resistance in ticks to Cypermethrin and Deltamethrin across the Abu Dhabi Emirate. There were few susceptible populations with zero resistance to Deltamethrin, indicating the need for the cautious use of acaricides. A lack of knowledge on the proper management and control of ticks, including the repetition of drug application in an irregular manner and improper doses of acaricides, results in the emergence of a resistant population of ticks. Improving the awareness about the effective management and control practices of tick-borne diseases in livestock is warranted as acaricide resistance is an emerging crucial animal health problem. Our study data show the threat of emerging acaricide resistance in the studied tick populations; further molecular studies are warranted to explore the genetic mutations underlying the resistance mechanisms in ticks. Since acaricide treatment failures are observed globally due to tick resistance, alternative strategies such as integrated pest management (IPM), including naturally resistant hosts, biological control using natural enemies, plant-based anti-tick formulations, and vaccine trials, are to be implemented. A multidisciplinary approach encompassing farmer education and training, regular surveillance and monitoring of tick resistance, policy-level changes governing drug use, and acaricide rotation strategies, as well as research into tick vaccines, can effectively mitigate acaricide resistance.

## Figures and Tables

**Table 1:** In vitro efficacy study of acaricides on engorged female ticks using the AIT-DD in three regions of the Emirate of Abu Dhabi. * resistant if %R is over 80%.

**a: Percentage resistance of *Hyalomma* species of ticks. Region—AL AIN.**											
**Drug**	Discriminating Dose (DD)										
Control	-	SiaSabra		Malaqet *		Sweihan	Qattara	AlSaad		Masaken	AlQuaa
		Camel	S&G	Camel	S&G	Camel	Camel	Camel	S&G	S&G	S&G
Cypermethrin	489.74 ppm	40	10	100 *	90 *	40	50	65	50	20	70
Deltamethrin	75 ppm	10	00	90 *	80 *	70	30	60	50	10	50
**b: Percentage resistance of *Hyalomma* species of ticks. Region—AL DHAFRAH.**											
**Drug**	Discriminating Dose (DD)										
Control	-	MZ Clinic I		Mirfa		Ghayathi		MZ Clinic II		MZ Clinic III	
		Camel		Camel		Camel		S&G		S&G	
Cypermethrin	489.74 ppm	76		50		50		70		60	
Deltamethrin	75 ppm	70		60		20		10		20	
**c: Percentage resistance of *Hyalomma* species of ticks. Region—ABU DHABI.**											
**Drug**	Discriminating Dose (DD)										
Control	-	Wathba Clinic 1				2		3		4	
		Camel		S&G		Camel		Camel		Camel	
Cypermethrin	489.74 ppm	40		40		30		20		40	
Deltamethrin	75 ppm	00		10		20		00		30	

S&G: sheep and goat.

**Table 2:** Association between the failure of tick treatment using acaricides and significant management factors in the final model using logistic regression.

Item	Level	OR	95% CI	*p* Value
Presence of vegetation around the farm	No	Ref	Ref	0.05
	Yes	2.23	−0.098–5.44	
Breed	Crossbred	Ref	Ref	0.018
	Indigenous	−3.58	(−7.31)–(−0.94)	

## Data Availability

All data are contained in the article; further enquiries can be made to the corresponding author.

## Supplementary Materials

The following supporting information can be downloaded at: https://www.mdpi.com/article/10.3390/vetsci12080712/s1.
