# Supplementary material for: In Vitro Detection of Acaricide Resistance in Hyalomma Species Ticks with Emphasis on Farm Management Practices Associated with Acaricide Resistance in Abu Dhabi, United Arab Emirates
Source: Vet Sci. 2025 Jul 29;12(8):712. doi: 10.3390/vetsci12080712 (PMC12390275; doi:10.3390/vetsci12080712)
Supplement: Supplementary file 1 [file vetsci-12-00712-s001.zip › vetsci-3681726-supplementary.pdf]

Description of the study population, farms characteristics and the management practices including their level and frequency are presented in Table.

| Item | Level | Frequency (%) |
|---|---|---|
| Region | Abu Dhabi | 6 (40.0) |
| | Al Ain | 4 (26.7) |
| | Al Zefrah | 5 (33.3) |
| Do you keep your animals together? | Yes | 13 (86.7) |
| | No | 2 (13.3) |
| Is there a vegetation around the farm? | Yes | 8 (53.3) |
| | No | 7 (46.7) |
| Where do you keep animals? | At home | 0 (00.0) |
| | On-farm | 15 (100.0) |
| How do external staff work on the farm? | Permanently | 14 (93.3) |
| | Temporary | 1 (6.7) |
| Acaricide application | Irregular | 11 (73.3) |
| | Regular | 4 (26.7) |
| What animal feeding method do you use? | Stall feeding | 13 (86.7) |
| | grazing | 0 (00.0) |
| | Mixed | 2 (13.3) |
| Tick infestation | Present | 15 (100.0) |
| | Absent | 0 (00.0) |
| Species | Small ruminant | 5 (33.3) |
| | Mix | 3 (20.0) |
| | Camel | 7 (46.7) |
| Breed | Indigenous | 5 (33.3) |
| | Crossbreed | 10 (66.7) |
| Do you have chronic tick infestation in your farm? | Yes | 13 (86.7) |
| | No | 2 (13.3) |
| Have you heard before about tick resistance? | Yes | 3 (20.0) |
| | No | 12 (80.0) |
| Are there stray animals (dogs/cates) on-the-farm infested with ticks? | Yes | 0 (00.0) |
| | No | 15 (100.0) |
| Were you aware that sandy floor is a risk factor for tick presence? | Yes | 6 (40.0) |
| | No | 9 (60.0) |
| In your view, which season is the highest risk for tick infestation? | Winter | 1 (6.7) |
| | Spring | 0 (00.0) |
| | Autumn | 0 (00.0) |
| | Summer | 14 (93.3) |
| When tick infestation is observed, what do you do? | Call veterinarian | 5 (33.3) |
| | Herbal/traditional Treatment | 8 (53.3) |
| | Sold their animals to reduce tick infestation | 2 (13.3) |
| Do you have a proper plan for acaricide use? | Yes | 0 (00.0) |
| | No | 15 (100.0) |
| Do you have proper disposal procedures or places for used acaricide bottles? | Yes | 0 (00.0) |
| | No | 15 (100.0) |

| | | |
|---|---|---|
| When was the last time you used acaricide? | Not more than 3 months ago | 12 (80.0) |
| | Not more than 6 months ago | 1 (6.7) |
| | less than 1 year ago | 2 (13.3) |
| What is your method of using acaricides? | Injection | 2 (13.3) |
| | Spraying/Dipping | 12 (80.0) |
| Do you treat animal houses and premises with acaricide? | Yes | 2 (13.3) |
| | No | 13 (86.7) |
| After how much time of using acaricides do you observe ticks at your farm? | Within months | 4 (26.7) |
| | 30 to 60 days later | 11 (73.3) |
| Have you experienced tick treatment failure? | Yes | 10 (66.7) |
| | No | 5 (33.3) |
| In case of treatment failure, what do you do? | Repeat the treatment | 4 (26.7) |
| | Increase the dose | 7 (46.7) |
| | Give new drug | 1 (6.7) |
| | Consult Veterinarian | 3 (20.0) |
| What is the best strategy to prevent tick infestation? | Biosecurity measures | 0 (00.0) |
| | Acaricide use | 8 (53.3) |
| | cannot be prevented | 7 (46.7) |
| What types of floors do you have? | Sandy | 15 (100.0) |
| | Concreted | 0 (00.0) |
| Can you identify the drugs used for tick treatment? | Cypermethrin | 6 (40.0) |
| | Ivermectin | 1 (6.7) |
| | Cypermethrin + Ivermectin | 8 (53.3) |
| Which drug is effective in your experience? | Cypermethrin | 1 (6.7) |
| | Ivermectin | 1 (6.7) |
| | Cypermethrin + Ivermectin | 13 (86.7) |
| Have your animals been diagnosed of any of these TickBorne diseases | Theileriosis | 14 (93.3) |
| | Babesiosis | 1 (6.7) |
| Have workers been bitten by ticks? | Yes | 7 (46.7) |
| | No | 8 (53.3) |
| What actions do you take for human tick bites? | Consult physician | 0 (00.0) |
| | Self-medication | 2 (13.3) |
| | No action | 13 (86.7) |
| Have you experienced and fever-like symptoms after tick bites? | Yes | 2 (13.3) |
| | No | 13 (86.7) |
